# A Dynamic Energy Budget Model for Kuruma Shrimp *Penaeus japonicus*: Parameterization and Application in Integrated Marine Pond Aquaculture

**DOI:** 10.3390/ani12141828

**Published:** 2022-07-18

**Authors:** Shipeng Dong, Dapeng Liu, Boshan Zhu, Liye Yu, Hongwei Shan, Fang Wang

**Affiliations:** 1 The Key Laboratory of Mariculture, Ministry of Education, Ocean University of China, 5 Yushan Road, Qingdao 266003, China; dongshipeng@stu.ouc.edu.cn (S.D.); mikeel@126.com (D.L.); zhuboshan@stu.ouc.edu.cn (B.Z.); yly12345678@yeah.net (L.Y.); shanhongwei@ouc.edu.cn (H.S.); 2 Function Laboratory for Marine Fisheries Science and Food Production Processes, Qingdao National Laboratory for Marine Science and Technology, 1 Wenhai Road, Qingdao 266003, China

**Keywords:** *Penaeus japonicus*, integrated marine pond aquaculture, Add-my-Pet, DEB theory, individual growth model

## Abstract

**Simple Summary:**

Individual growth models of animals are the basis of ecosystem models, which are important tools for aquaculture management. The lack of mechanistic growth model studies of kuruma shrimp, an important integrated marine aquaculture species, has hindered ecosystem-level aquaculture management. In this study, an individual growth model of kuruma shrimp was constructed based on the dynamic energy budget theory, and the goodness of fit of both parameterization and application was high. The results showed that the model could reproduce the growth of kuruma shrimp in terms of total length and wet weight.

**Abstract:**

Individual growth models can form the basis of population dynamics assessment and ecosystem model construction. In order to provide a basic module for an ecosystem model of an integrated marine aquaculture pond, an individual growth model was constructed for kuruma shrimp (*Penaeus japonicus*) based on dynamic energy budget (DEB) theory. The model was first parameterized based on a covariation method using the Add-my-Pet (AmP) procedure. The parametric estimation model underestimated the ultimate abdominal length for female shrimp, and the predicted values of other zero-variate parameters were generally consistent with observed values. The relative errors of the predicted and observed values of the univariate data set within three geographical regions showed acceptable goodness of fit. Parameter estimation achieved an overall goodness of fit with a mean relative error of 0.048 and a symmetric mean squared error of 0.066. A DEB model was constructed using the estimated parameters, and the goodness-of-fit indicators (R square, mean bias and absolute and relative root mean square error) showed that the model was able to reproduce the growth of kuruma shrimp in terms of total length and wet weight with high accuracy. The results provide data to support the subsequent development of integrated aquaculture management at the ecosystem level.

## 1. Introduction

As a typical integrated multitrophic aquaculture (IMTA) model, integrated marine pond aquaculture has the advantages of increased feed efficiency, high-quality product, high yield and low pollution emissions compared with extensive, semi-intensive and intensive aquaculture models [1]. In China, integrated marine pond aquaculture is dominated by shrimp and crab species, forming integrated aquaculture models, such as shrimp–crab, shrimp–crab–shellfish, shrimp–crab–fish and shrimp–crab–shellfish–fish [2]. Owing to their strong vitality, high quality meat and high commercial prices, kuruma shrimp (*Penaeus japonicus*) are popular among farmers, with an average annual production of more than 50,000 tons in China in the past decade, making kuruma shrimp an important integrated marine aquaculture species [3]. As an efficient aquaculture ecosystem, integrated marine pond aquaculture has been studied in depth in terms of economic benefits, disease prevention and control and water quality regulation [4,5], but further application and promotion requires theoretical research at the ecosystem level to support aquaculture planning and management [6].

The core of aquaculture management based on ecosystem models is to reproduce environmental dynamics through mathematical functions in order to simulate scenarios such as different species pairings and different aquaculture practices with the aim of maximizing economic and ecological benefits [7,8]. Individual growth models are key submodules of ecosystem models. Individual growth models of crustaceans mainly include two types of models: simple empirical growth models [9,10] and mechanism-based growth models [11].

Empirical growth models, in which empirical data are collected through experiments or surveys and statistically regressed, are more region-specific, and further applications are usually integrated into other empirical models [12]. Mechanism-based growth models describe the material or energy-partitioning processes of individuals through a set of complex differential equations that vary in time and/or space, and their simulation results can be validated with observations. Although the fit may be lower than that of empirical growth models, they can be incorporated into larger ecosystem models [13]. Currently, the most widely used mechanistic models of individual growth include the SFG model, which is based on the scope for growth concept [14], and the DEB model, which is based on dynamic energy budget theory [15].

Since the DEB theory was first proposed by S.A.L.M Kooijman [16], more than 1000 papers related to the DEB model have been published, including applications in various IMTA ecosystems [17,18,19,20]. The DEB model has achieved good results in studies on northern krill (*Meganyctiphanes norvegica*), brown shrimp (*Crangon crangon*) and Chinese shrimp (*Fenneropenaeus chinensis*), providing information to support the assessment and management of fishery resources [21,22,23]. 

The accurate acquisition of DEB parameters, which reflect the differences in life history and energetics among species, is a prerequisite for model construction [24]. The traditional approach to DEB model parameterization is to obtain data sets based on factorial design experiments with controlled conditions, such as ingestion, oxygen consumption and starvation experiments [25,26]. However, the AmP (Add-my-Pet) project and the creation of the associated database and procedure have standardized and improved DEB parameter estimation methods [27]. The ability of the AmP procedure to extract DEB parameters from animals with less critical data has effectively facilitated the development and application of DEB models [28].

In this study, a kuruma shrimp DEB model was parameterized using the AmP procedure, and the growth of kuruma shrimp in a marine integrated aquaculture pond was simulated using the model. The model will provide the basic module and data support for subsequent construction of ecosystem models for integrated marine aquaculture ponds.

## 2. Materials and Methods

### 2.1. DEB Model

The DEB model used in this study is based on the κ rule to describe the absorption, reserve dynamics and utilization of energy by individuals [16]. The DEB model equations developed for kuruma shrimp are presented in Table 1. Simulation experiments and statistical analyses were performed in Python 3.7.

**Figure 1:**
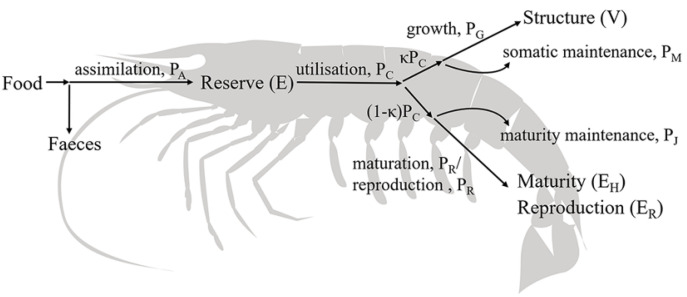
Schematic representation of the parameters used to describe kuruma shrimp in a standard DEB model.

Within the model, individuals are described by four state variables: structure (V), reserve (E), reproduction (E_R_) and maturity (E_H_) (Figure 1). Energy from the environment is first incorporated into the reserve; then, part of it is used for growth and maintenance, and another part is used for maturity and reproduction [29].

Individuals may lose weight or die if reserves are not sufficient to sustain the consumption of normal activities. They obtain energy from food and, which is transferred to reserves through the assimilation process (P_A_). The reserve is subsequently metabolized (P_C_) to be used for growth (P_G_), maintenance (P_M_) and maturity/reproduction (P_R_). A constant fraction (κ) of the mobilized reserve is allocated to somatic tissue, which includes somatic maintenance and growth, whereas the remaining 1-κ fraction, after subtracting the costs associated with maturity maintenance (P_J_), is used for maturity/reproduction.

### 2.2. Parameter Estimation

The parameters of the kuruma shrimp DEB model were estimated according to the Add-my-Pet (AmP) procedure, which is based on the improved version of the covariance method, i.e., the parameters are continuously adjusted by minimizing the loss function to achieve a reduction in the difference between the observations and predictions [27]. Loss function minimization is achieved by an automatic execution check of the Nelder–Mead simplex method [30]. The new procedure uses mean relative error (MRE) and symmetric mean squared error (SMSE) to assess the overall goodness of fit [28]. The data needed for parameterization include zero-variate data, univariate data and pseudo-data, among which zero-variate data and univariate data are extracted from existing studies and records [31]. The DEB parameters of kuruma shrimp were obtained by running the AmP procedure in Matlab R2016a.

Zero-variate data related to the biological features of kuruma shrimp were mainly obtained from the records of Song et al. [32]. Kuruma shrimp have a short life cycle and grow rapidly, with a maximum life span of 2 years. Kuruma shrimp is a subtropical species with strong adaptability to temperature, although changes in temperature still have a significant effect on its growth and developmental time. At water temperatures of 22–29 °C, fertilized eggs take 13 to 20 h to develop and hatch into the nauplius stage. After a further 36 h, the nauplius stage develops into the protozoa stage, and individuals start to feed. Sexual differentiation of the gonads is normally observed on approximately the 60th day after protozoa become juvenile shrimps [33]. The ultimate length of a male shrimp is approximately 17 cm, and that of female shrimp is approximately 27 cm, with an ultimate wet weight of 130 g [34]. The maximum annual egg production of kuruma shrimp is about 700,000, with an average of 500,000 to 600,000 [35,36].

Univariate data (growth curves in length and weight) were obtained from three geographic areas along the Chinese coast: two bays (Guzhenkou Bay and Laoshan Bay) [37,38] and one intensive aquaculture pond in Hainan Province, China [39]. Annual shrimp stock enhancement is carried out in late June and early July in the two bays. During the periods from August to October 2006 and 2007, surveys were conducted every half month in Guzhenkou Bay, with each survey measuring total body length and wet weight of female and male shrimp. Data from a total of 11 surveys over two years were combined in the AmP procedure. Seven semi-monthly surveys were also conducted in Laoshan Bay from August to October 2008. At least 30 shrimp were collected in each survey in the two bays (average water temperature: 22 °C). In the intensive aquaculture pond, the total length and wet weight of 30 shrimp were recorded every 10 days during a 100-day aquacultural experiment from August to December 2009 (average water temperature: 28 °C).

Growth, ingestion and metabolism of individuals are affected by temperature, and the response of physiological rates to temperature in the DEB model follows an Arrhenius relationship [29]. Arrhenius temperature (T_A_) is usually determined by measuring the effect of temperature on oxygen consumption or other physiological rates, and existing studies on the variation of oxygen consumption rate with temperature in kuruma shrimp have mainly focused on the juvenile stage [32,40,41].

In this study, parameters of similar species, such as northern krill, brown shrimp and Chinese shrimp [21,22,23], were combined, and T_A_ was set at 6200 K. The remaining four parameters (T_L_, T_H_, T_AL_ and T_AH_) used to describe the temperature tolerance range of enzyme activity were manually adjusted to provide the best fit [42].

### 2.3. Integrated Aquaculture Pond Experimental Setup

An integrated aquaculture experiment was conducted in a marine pond in Zhoushan City, Zhejiang Province, China (24°35′ N, 112°7′ E), containing swimming crab (*Portunus trituberculatus*), kuruma shrimp (*P. japonicus*) and razor clam (*Sinonovacula constricta*). The pond area was 1.33 ha, with an average water depth of 1.2 m during the experimental period. Kuruma shrimp with total lengths of 1.02 ± 0.07 cm and an average wet weight of 8.7 × 10^−3^ g were stocked to a density of 22.5 × 10^4^ ind·ha^−1^. The experimental period ran from June 2020 to November 2020—a total of 180 days.

Water temperature and food density are the forcing functions used to run the model. Water temperature was continuously recorded by a water temperature recorder (HOBO-MX2201, America) [43]. The nutrients required for shrimp and crab growth were provided by feeding with iced trash fish. Growth data, including total length and wet weight, were measured every 15 days. DEB theory describes individual feeding using a Holling type-II functional response; the rates of feeding and energy assimilation from the environment are proportional to food density (i.e., *f* = X/(X_K_ + X), where X is the food density, and X_K_ is the half-saturation constant, 0 ≤ *f* ≤ 1 [44]). In the absence of information on biological feeding parameters or food biomass, *f* can be obtained based on the model fit [45]. In DEB models for animals artificially fed feedstuff, *f* is usually set to a constant [46].

## 3. Results

### 3.1. Model Parameters

The completeness of the data used for parameter estimation was set at 4.2 according to the criteria developed by Lika et al. [31]. Zero-variate predictions of the kuruma shrimp DEB model were completed using the AmP procedure, and a comparison of predicted and observed values is presented in Table 2. The model underestimated the ultimate abdominal length for female shrimp, whereas the predicted values of other parameters were generally consistent with the observed values.

**Figure 2:**
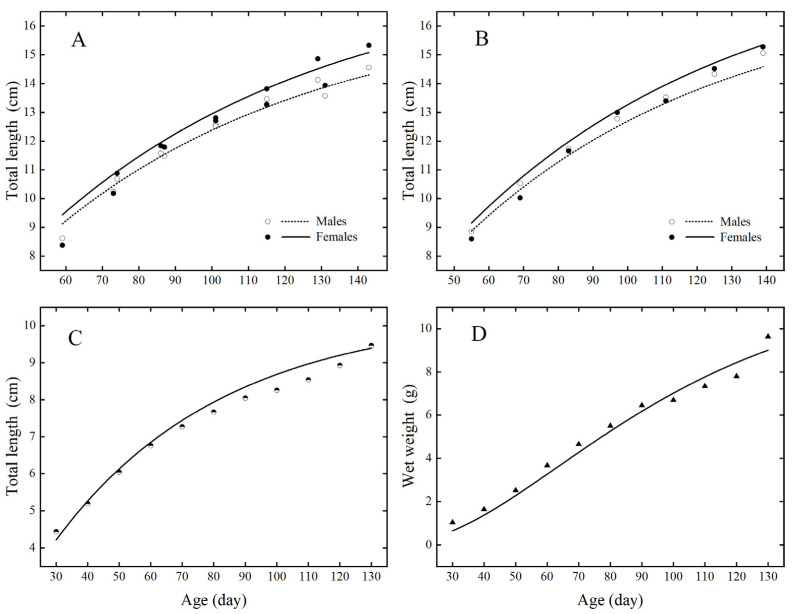
Comparison of model predictions and observations of total length and weight in Guzhenkou Bay (**A**), Laoshan Bay (**B**) and an intensive aquaculture pond Hainan Province, China (**C**,**D**). The lines indicate model predictions of growth. The circles and triangles indicate observations of total length and wet weight growth, respectively. In (**A**,**B**), solid lines and symbols are predictions for females, and dashed lines and open symbols are predictions for males. The observations reflect the mean growth of the species in each environment.

Univariate simulation results for the kuruma shrimp DEB model in the three environments from which the model parameters were derived are shown in Figure 2. For both bays, simulated values of total length were greater than those observed for male shrimp from day 60 to day 140. The initial simulated values of male and female shrimp were slightly overestimated in Guzhenkou Bay, with relative errors (RE) of 0.0297 and 0.0170 for simulated and observed values of female and male shrimp, respectively. In Laoshan Bay, the simulated values of female shrimp were all greater than the observed values, the simulated values of male shrimp were all less than the observed values, and the relative errors of simulated and observed values of female and male shrimp were 0.0279 and 0.0199, respectively. In the intensive aquaculture pond, the prediction and observation of total length and wet weight were close, and the relative errors of predicted and observed values were 0.0299 and 0.0735, respectively.

The goodness of fit for the zero and univariate parameter estimates was generally acceptable, with an MRE of 0.048 and a SMSE of 0.066. The estimated parameters of the kuruma shrimp DEB model are presented in Table 3.

### 3.2. Model Application

**Figure 3:**
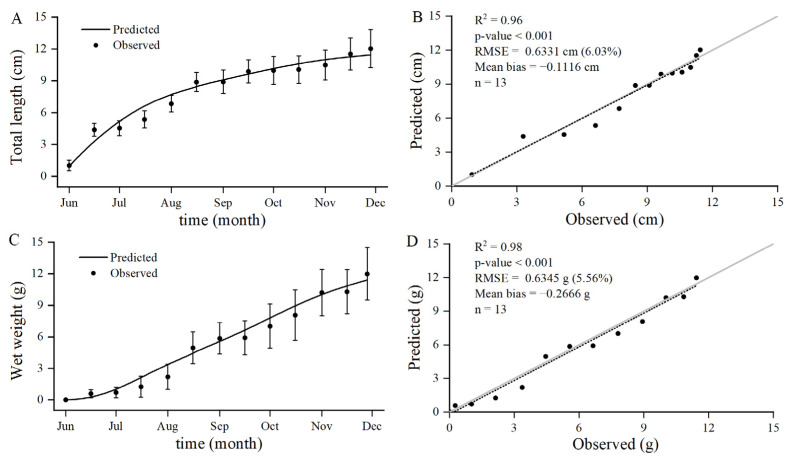
Modelled (lines) and observed (circles with bars for standard deviation) growth in terms of total length (**A**) and wet weight (**C**) for kuruma shrimp in an integrated marine aquaculture pond, and validation for total length (**B**) and wet weight (**D**) growth modeled with several goodness-of-fit indices.

The observed and modelled growth of kuruma shrimp in the experimental integrated aquaculture pond from June to November 2020 are shown in Figure 3. The optimal fit of the model to the observation resulted in an *f* value of 0.62. According to the modelled values, in the first 2 months, kuruma shrimp grew rapidly, and the modelled values were slightly higher than the observed values. From August to October, the shrimp entered a slow growth period, as demonstrated by both simulated and observed values. After October, the growth rate of the shrimp picked up, and modelled and observed values matched more closely.

Statistical tests were performed using coefficient of determination (R^2^), root mean square error (RMSE) and mean bias (MB) for the goodness of fit of the model. Compared with the observed results, the RMSEs of total length (0.9342~11.4362 cm) and wet weight (0.0082~11.4216 g) were 0.6331 cm (6.03%) and 0.6345 g (5.56%), respectively, and the MBs were −0.1116 cm and −0.2666 g, respectively. The modelled values were in general agreement with the observed values, indicating that the model could simulate and reflect the changes of total length and wet weight.

## 4. Discussion

In this study, an individual growth model of kuruma shrimp in an experimental integrated marine aquaculture pond was constructed based on DEB theory, and the parameterization and simulation results all showed good fits. We hope that this will form the basis for an ecosystem model for integrated aquaculture ponds. DEB theory has been widely used for aquatic animals, such as fish, shellfish and crustaceans [23,48,49]. However, this is the first time this theory has been applied to kuruma shrimp.

In this study, iced trash fish was the common food for both the kuruma shrimp and the main feeder in the integrated experimental aquaculture system, *P. trituberculatus*. Although feeds were excessive, unlike the optimal food condition (*f* = 1) in a previous DEB model for *P. trituberculatus* [43], the modelled results for kuruma shrimp fit better with the observed results when *f* was 0.62, probably because feed palatability (composition, shape, size, etc.) was optimized for *P. trituberculatus*.

Animal species in DEB theory use the same model structure, and the differences in their life histories are reflected by the differences in their DEB parameter sets [50]. Currently, DEB model parameter estimates are mainly obtained through physiological experiments and the covariation method, which links the parameters to experimental and field observations. [51]. DEB parameterization experiments are laborious, and some parameters cannot be measured directly but still need to be obtained by “free fitting” [52]. AmPtool can be downloaded directly from https://www.bio.vu.nl/thb/deb/deblab/add_my_pet (accessed on 1 March 2009). The AmP method has a wide range of search parameters and good substitutability of the required parameters and has become a reliable method for estimating DEB model parameters after several optimizations [28,53]. The diversity of environmental conditions may produce individual differences, and artificial management (strain, feeds, oxygenation, water quality regulation, etc.) may increase such differences [54]. Despite the small differences in parameter values between individuals of the same species, biological data from certain long-established or characteristic strains were not selected from data collection in this study to reduce possible parameter errors [55].

The molting process in crustaceans leads to discontinuities in growth, which makes individual growth models difficult to apply to crustaceans. Two key components of crustacean molt dynamics (molt increment, MI and intermolt period (IP)) are necessary for the construction of discontinuous growth models [12]. Many simple empirical growth models have been developed to model crustacean discontinuous growth by statistically analyzing MI and IP data to relate the molting process to pre- and post-molt size [12,56]. Mechanism-based growth models for simulating discontinuous growth processes in crustaceans require suitable trait parameters as thresholds. Currently, the molting process is mostly ignored in the SFG and DEB growth models of shrimp [14,23]. In discontinuous growth models for crab based on DEB theory, single and multiple molts have been simulated by two parameters: carbon mass/wet mass and condition factor, respectively [11,43]. We applied these two parameters to the discontinuous growth model to simulate the molting behavior of kuruma shrimp, but they were not successful.

DEB models do not provide population dynamics directly but can provide detailed information on quantified physiological processes and bioenergetics, which can then be extended to populations using their dynamics resulting from interactions between individuals under dynamic environmental conditions [54]. Unlike bay and deep-sea aquaculture, the physical processes involved in pond aquaculture are relatively simple, which makes biological processes more critical in predictive models [57]. The kuruma shrimp DEB model developed here will provide an important component for ecological models that aim to simulate the population dynamics of integrated marine aquaculture ponds and provide assessments of their carrying capacity.

## 5. Conclusions

In this study, the parameters of the DEB model for kuruma shrimp were estimated using the AmP method, and zero-variate, univariate and overall goodness-of-fit analyses (RE, MRE and SMSE) showed the reasonableness of the estimated parameters. A DEB model for kuruma shrimp in an experimental integrated marine aquaculture pond was constructed using the estimated parameters. Statistical tests of simulated and observed values showed that the model could simulate the growth of total length and wet weight of kuruma shrimp. This study can be used not only for individual growth prediction but also coupled with ecosystem models to provide important information for carrying capacity and environmental pollution assessment at the ecosystem level in integrated aquacultural systems.

## Figures and Tables

**Table 1:** Equations describing the energy fluxes in the standard DEB model for kuruma shrimp.

Definition	Equation
Temperature dependence	$k\left(T\right)=k_{0}\exp\left\{\frac{T_{A}}{T_{0}}-\frac{T_{A}}{T}\right\}{\left(1+\exp\left\{\frac{T_{\mathrm{AL}}}{T}-\frac{T_{\mathrm{AL}}}{T_{L}}\right\}+\exp\left\{\frac{T_{\mathrm{AH}}}{T_{H}}-\frac{T_{\mathrm{AH}}}{T}\right\}\right)}^{-1}$
Assimilation rate	$P_{A}=\;k\left(T\right)\cdot f\cdot \left\{P_{\mathrm{Am}}\right\}\cdot V^{2/3}$
Catabolic rate	$P_{C}=k\left(T\right)\frac{\frac{E}{V}}{\left[E_{G}\right]+\kappa \cdot \frac{E}{V}}\left(\frac{\left[E_{G}\right]\cdot \left\{P_{\mathrm{Am}}\right\}\cdot V^{\frac{2}{3}}}{\left[E_{M}\right]}+\left[P_{M}\right]\cdot V\right)$
Maintenance rate	$P_{M}=k\left(T\right)\cdot \left[P_{M}\right]\cdot V$
Maturity maintenance rate	$P_{J}=k\left(T\right)\cdot \min\left(V,V_{P}\right)\cdot \left[P_{M}\right]\cdot \left(\frac{1-\kappa }{\kappa }\right)$
Reserve dynamics	$\frac{\mathrm{dE}}{\mathrm{dt}}={\;P}_{A}-P_{C}$
Reproductive reserve dynamics	$\frac{{\mathrm{dE}}_{R}}{\mathrm{dt}}=\left(1-\kappa \right)P_{c}-P_{J}$
Biovolume growth	$\frac{\mathrm{dV}}{\mathrm{dt}}=\frac{\kappa \;\cdot P_{C}-P_{M}}{\left[E_{G}\right]}$
Volume	$V=\frac{E_{V}}{\left[E_{G}\right]}$
Total length	$L=\frac{V^{1/3}}{\delta_{m}}$
Wet weight	$\mathrm{Ww}=\frac{E}{\mu_{E}}+\frac{\kappa_{R}\cdot E_{R}}{\mu_{E}}+V\cdot \rho$

**Table 2:** List of zero-variate data used to estimate parameters of the kuruma shrimp DEB model. References of observed data and relative error (RE) are specified. The average temperature at which parameters were measured is provided.

Symbol	Unit	Observation	Prediction	Parameter	RE	References
*a_b_*	d	2.00	2.05	age at birth (27 °C)	0.0277	[32]
*a_m_*	d	730	728	life span (22 °C)	0.0018	[32]
*t_p_*	d	70.00	70.03	time since birth at puberty (26 °C)	0.0005	[32,33]
*L_i_*	cm	27.00	19.83	ultimate abdominal length for female shrimp	0.2656	[34]
*L_im_*	cm	17.00	18.46	ultimate length for male shrimp	0.0861	[34]
*Ww_i_*	g	130.0	135.9	ultimate wet weight	0.0457	[34]
*R_i_*	#d^−1^	1507	1510	ultimate reproduction rate (27 °C)	0.0023	[36,47]

**Table 3:** Parameter values of the DEB model for kuruma shrimp at a reference temperature of 20 °C.

Symbol	Value	Unit	Parameter	Source
z	0.76	-	zoom factor for female shrimp	This study
$z_{m}$	0.71	-	zoom factor for male shrimp	This study
{F_m_}	6.5	J·cm^−2^·d^−1^	maximum specific searching rate	This study
ύ	0.0336	cm·d^−1^	energy conductance	This study
[E_G_]	4439	J·cm^−3^	volume-specific costs for structure	This study
[E_M_]	13,235	J·cm^−3^	maximum storage density	This study
κ	0.98	-	fraction of catabolic flux to growth and maintenance	This study
$\kappa_{R}$	0.95	-	fraction of reproductive reserves	This study
δ_M_	0.1585	-	shape coefficient	This study
{P_Am_}	1823	J·cm^−2^·d^−1^	maximum surface-area-specific assimilation rate	This study
[P_M_]	569	J·cm^−3^·d^−1^	volume-specific maintenance rate	This study
T_1_	293	K	reference temperature	[32]
T_A_	6200	K	Arrhenius temperature	[21,22,23]
T_H_	302	K	upper boundary temperature of the tolerance range	[32]
T_L_	283	K	lower boundary temperature of the tolerance range	[32]
T_AH_	33,800	K	Arrhenius temperature for the rate of decrease at upper boundary	[32,40,41]
T_AL_	13,300	K	Arrhenius temperature for the rate of decrease at lower boundary	[32,40,41]
ρ	1	g·cm^−3^	volume-specific dry flesh weight	This study
$E_{\mathrm{Hb}}$	0.0013	J	maturity at birth	This study
$E_{\mathrm{Hj}}$	0.0966	J	maturity at metamorphosis	This study
$E_{\mathrm{Hp}}$	2349	J	maturity at puberty	This study
$h_{a}$	1.8 × 10^−7^	J	Weibull aging acceleration	This study
$s_{G}$	0.0001	-	Gompertz stress coefficient	This study

## Data Availability

The data presented in this study are available in the article. Further information is available upon request from the corresponding author.
